# Development of a Multi-Channel Ultra-Wideband Electromagnetic Transient Measurement System

**DOI:** 10.3390/s25041159

**Published:** 2025-02-14

**Authors:** Shaoyin He, Xiangyu Chen, Bohao Zhang, Liang Song

**Affiliations:** State Key Laboratory of Electrical Insulation and Power Equipment, School of Electrical Engineering, Xi’an Jiaotong University, Xi’an 710049, China; 1037889260@stu.xjtu.edu.cn (X.C.); zbh3121104133@stu.xjtu.edu.cn (B.Z.); 450406291@stu.xjtu.edu.cn (L.S.)

**Keywords:** transient electric field measurement, ultra-wideband electromagnetic pulse, electric field probe, field programmable gate array

## Abstract

In complex electromagnetic environments, such as substations, converter stations in power systems, and the compartments of aircraft, trains, and automobiles, electromagnetic immunity testing is crucial. It requires that the electric field sensor has features such as a large dynamic measurement range (amplitude from hundreds of V/m to tens of kV/m), a fast response speed (response time in the order of nanoseconds or sub-nanoseconds), a wide test bandwidth (DC to 1 GHz even above), miniaturization, and robustness to strong electromagnetic interference. This paper introduces a multi-channel, ultra-wideband transient electric field measurement system. The system’s analog bandwidth covers the spectrum from DC and a power frequency of 50 Hz to partial discharge signals, from DC to 1.65 GHz, with a storage depth of 2 GB (expandable). It overcomes issues related to the instability, insufficient bandwidth, and lack of accuracy of optical fibers in analog signal transmission by using front-end digital sampling based on field-programmable gate array (FPGA) technology and transmitting digital signals via optical fibers. This approach is effectively applicable to measurements in strong electromagnetic environments. Additionally, the system can simultaneously access four channels of signals, with synchronization timing reaching 300 picoseconds, can be connected to voltage and current sensors simultaneously, and the front-end sensor can be flexibly replaced. The performance of the system is verified by means of a disconnect switch operation and steady state test in an HVDC converter station. It is effectively applicable in scenarios such as the online monitoring of transient electromagnetic environments in high-voltage power equipment, fault diagnosis, and the precise localization of radiation sources such as partial discharge or intentional electromagnetic interference (IEMI).

## 1. Introduction

Electromagnetic radiation fields are present during processes such as lightning strikes, high-voltage switch operations, static electricity build-up, and partial discharge caused by insulation damage. These electromagnetic radiation fields are characterized by high intensity, rapid variation, and a broad frequency spectrum, and they can couple into electronic systems through antennas, apertures, cables, and other paths [1,2], potentially causing temporary or permanent damage to various electronic devices and systems [3,4,5,6]. The measurement of transient electromagnetic fields is a crucial aspect of electromagnetic field protection research. It plays a vital role in studying the propagation characteristics of transient electromagnetic fields, developing protection methods, and evaluating their effects.

Currently, there are two main approaches to measuring a transient electric field—the first one is based on the photoelectric effect, where the electric field signal is converted into an optical signal for measurement. However, optical electric field sensors have complex manufacturing processes, are significantly affected by external environmental factors such as temperature, and have a limited dynamic range, which restricts their widespread application.

The other approach involves electric field sensors based on the principle of electromagnetic induction. Such sensors have been applied previously in the field of EMP measurement, and the related measuring technology is more mature. Baum and others systematically summarized nuclear EMP measurement technology [7]. As EMP measurement efforts have progressed, the International Electrotechnical Commission has successively formulated and published a series of international standards related to HEMP measurements, including radiation interference, conduction interference, immunity, and high-power transient parameter measurements [8,9,10,11]. Subsequently, Montena Ltd. (Rossens, Switzerland) commercialized the production of gradual conical dipole antennas, with the parameters of their products adjusted according to different frequency requirements, reaching an upper cutoff frequency of 10 GHz. In 2007, Koshelev and others from the Russian Institute of High-Current Electronics designed a two-dimensional active electric dipole antenna for measuring ultra-wideband pulses, with a working bandwidth of 0.6 to 4.6 GHz [12]. Baum et al. proposed a differential electric field sensor known as D-dot for EMP electric field signal measurement [13]. The output voltage signal of the sensor is proportional to the differential of the electric field, making it suitable for ultrafast pulse signal measurement. However, this sensor requires integration of the output signal, which is suitable in the low-SNR and -frequency scenario. Ref. [14] proposes an ultra-wideband time-domain antenna coupling electric-field energy through a pair of differential dipoles, with operating frequency range of 10 MHz~1 GHz.

For measuring the transient electromagnetic environment in power system substations, a nanosecond-level transient electric field measurement system has been developed, with a bandwidth range of 1 kHz to 460 MHz [15]. Refs. [16,17,18,19] explored the frequency characteristics of the radiated electromagnetic fields generated by substation switching operations and their coupling effects on secondary equipment. Their results indicate that the amplitude of the transient radiated electromagnetic fields generated by high-voltage switches ranges from hundreds of V/m to tens of kV/m, with spectral components spanning from several hundred kHz to several hundred MHz.

With the deepening research in electromagnetic compatibility, electromagnetic interference, and protection technologies within power systems, measurement systems for transient electromagnetic fields in power systems are required to have the following characteristics: (1) sufficient bandwidth; (2) good sensitivity across different amplitude levels due to the signal under test potentially varying significantly in amplitude; (3) a compact and miniaturized size which should not affect the electromagnetic environment being measured; and (4) effective shielding, grounding, and filtering measures to suppress common-mode interference [20].

The current transient electric field measurement systems experience issues such as insufficient bandwidth, limited precision, and unstable amplitude coefficients. In the electromagnetic environment of power systems, the spectrum of steady-state signals can be as low as 50 Hz or even lower. However, existing electric field measurements often focus on improving high-frequency performance, making them ineffective for measuring low-frequency signals. In power grids, switching operations of power electronics such as IGBTs can produce spectral components in the range of several kHz or even lower. The current measurement systems struggle to effectively address both low-frequency and high-frequency electromagnetic interference signals [21].

To achieve interference-resistant measurement in complex, strong electromagnetic environments, fiber optic technology is widely used [21,22]. However, after multiple field applications, it has been found that directly transmitting analog signals through fiber optic links presents the following drawbacks: (1) The analog bandwidth is limited by the fiber optic transmitter and receiver, with the low-frequency cutoff generally only reaching as low as around 1 kHz, and achieving a high-frequency cutoff of 600 MHz or above is very challenging [15]. (2) The transient electric field signals in the environment under test have a very wide amplitude range, from tens of V/m to the kV/m level, or even higher. Within this range, the conversion accuracy of the fiber optic link for signals with smaller amplitudes is poor and unstable, leading to significant measurement errors and even making it difficult to determine the effective amplitude.

With the development of high-speed data acquisition technology, particularly advancements in analog-to-digital converter (ADC) and field-programmable gate array (FPGA) technologies, digital measurement technology has become possible. Digital measurement technology allows for long-distance digital data transmission, making data easier to store and process, and can capture transient signals with high precision. In the UK, Mohamed et al. developed a novel time-based triggering logic implemented in FPGA, which is used for locating partial discharges in power cables [23]. Russer et al. developed a real-time operating time-domain electromagnetic interference measurement system based on data acquisition and digital signal processing technology, applied in the frequency range of 30 MHz to 1 GHz [24,25]. However, it cannot satisfy the measurement requirements for power systems in the low-frequency band. Cheng et al. designed a pulse signal data acquisition system based on FPGA technology. This system uses fiber optics to directly transmit digital signals, solving the problem of signal distortion in long-distance transmission of analog signals through cables in nuclear explosion pulse beam measurements [26]. Thus, it is evident that digital measurement technology offers certain advantages over traditional analog measurement techniques in terms of signal measurement, transmission, and processing.

To the best of the authors’ knowledge, no studies have been reported on multi-channel measurement systems designed for ultra-wideband electromagnetic transient measurements which achieve time-sequence synchronization across multiple measurement channels. Such systems are in high demand for a wide range of critical applications, including locating and detecting transient electromagnetic radiation sources such as partial discharge or intentional electromagnetic interference (IEMI), state sensing of power equipment, fault monitoring and diagnostics based on multi-physical transient signals involving electromagnetic fields, overvoltage, and overcurrent, etc.

**Figure 1:**
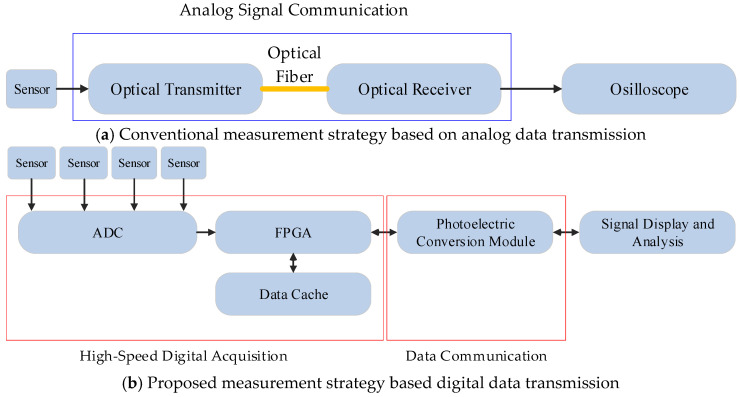
The measuring strategy comparison between conventional analog data transmission and digital data transmission.

Conventional measurement strategies transmit analog signals via optical fibers, which face issues such as unstable amplitude coefficients leading to large measurement errors, weak electromagnetic interference resistance of analog signals, and limited bandwidth due to the constraints of the optoelectronic conversion module. In contrast, this paper recommends digitizing the analog signal at the output of the sensor, i.e., at the front end of the measurement system, and transmitting the digital signal through an optical fiber link. This approach not only enhances interference resistance but also effectively overcomes the bandwidth limitation and unstable amplitude coefficient problems imposed by the optoelectronic devices. The comparison of measuring strategies and the schematic diagram are shown in Figure 1.

This paper focuses on the measurement issues of broadband transient electric field signals and studies electromagnetic pulse measurement technology based on high-speed digital sampling techniques using FPGA. The main research content includes the following aspects: Section 2 explores the design of a miniaturized transient electric field sensor based on electrically small monopole antenna and develops corresponding ultra-wideband conditioning circuits. Section 3 introduces the local high-speed digital sampling technology based on FPGA, enabling the digital sampling of measured analog signals. The time-domain calibration of the transient electric field measurement system is illustrated in Section 4. Section 5 carries out experimental validation in a power system converter station. Conclusions and discussions are presented in Section 6.

## 2. Electric Field Sensor

Monopole antennas are widely used in electromagnetic interference testing, electromagnetic compatibility assessments, and electromagnetic environment measurements. When the electric field component of an electromagnetic wave is present around the antenna, it induces a voltage along the axial direction of the antenna, thereby sensing the electric field in the environment. When the antenna satisfies the electrically small condition (where the geometric size is much smaller than the shortest wavelength in the signal), the time for the signal to propagate through the antenna is much shorter than the rise time of the leading edge of signal, thus preserving the original shape of the pulse. By using an electrically small antenna, which is largely independent of frequency, as a receiving antenna to detect transient pulse electric fields, the time-domain waveform of the incident electric field can be measured with minimal distortion. Additionally, the antenna has the characteristic of omnidirectional reception of electromagnetic fields in the horizontal plane [22].

The design of an electric field measurement probe primarily consists of the monopole antenna, the signal conditioning circuit, and a metal shielding case. The monopole antenna converts electromagnetic field signals in space into electrical signals. The output signal power of a monopole electrically small antenna is limited, making it unsuitable for transmission via coaxial cables. Therefore, a high-impedance input of an operational amplifier is used to achieve impedance transformation and matching for the antenna. The metal shielding case encloses the internal circuit board, serving both as a shield and as a mirror ground plane for the antenna.

### 2.1. Principle of a Monopole Electrically Small Antenna

An electrically small antenna operates under the condition h≪λ/2π, where h is the antenna height and λ is the operating wavelength. It has advantages such as a small geometric size, a wide bandwidth, an adjustable and large measurement range, omnidirectional measurement, and adjustable parameters, meaning that it is commonly used for measuring transient electric field signals. In this regime, the monopole antenna primarily functions as a capacitive element, responding to the displacement current induced by the incident electric field. The induced voltage V across the antenna terminals is proportional to the effective height $h_{e}$ and the local electric field E:

(1) $$V(t)=h_{e}\cdot E(t),h_{e}\approx \frac{h}{2}$$

The sensitivity of the rod antenna can be adjusted by varying its height *h* to accommodate different measurement environments. To ensure a wideband frequency response, the antenna can be modeled as a parallel RC circuit, where $C_{a}$ represents the antenna capacitance and $R_{l}$ is the load impedance of the measurement system. The transfer function H(s) of the system is given by

(2) $$H(s)=\frac{V(s)}{E(s)}=\frac{sR_{l}C_{a}h_{e}}{1+sR_{l}C_{a}}$$

From this, the lower cutoff frequency $f_{L}$ can be expressed as

(3) $$f_{L}=\frac{1}{2\pi (C_{l}+C_{a})R_{l}}$$

To extend the low-frequency response (e.g., down to 50 Hz), a high-impedance buffer circuit is employed to maximize $R_{l}$, ensuring accurate measurements of both steady-state and transient signals. The higher cutoff frequency $f_{h}$ is limited by the size of the antenna, which is determined by the condition of h≪λ/2π.

Compared to traditional D-Dot sensors, which measure the time derivative of the electric field and require numerical integration, the monopole antenna directly measures the electric field strength E(t). This simplifies the signal processing chain while maintaining a wideband response, making it particularly suitable for capturing sub-nanosecond transients and low-frequency steady-state components simultaneously.

Practical design considerations include minimizing parasitic capacitance and inductance to extend the high-frequency response and optimizing the antenna geometry to balance sensitivity and physical size. These principles ensure that the monopole antenna achieves a flat response across DC to GHz frequencies, enabling precise transient electromagnetic measurements in power system applications.

### 2.2. Simulation of the Electric Field Sensor

**Figure 2:**
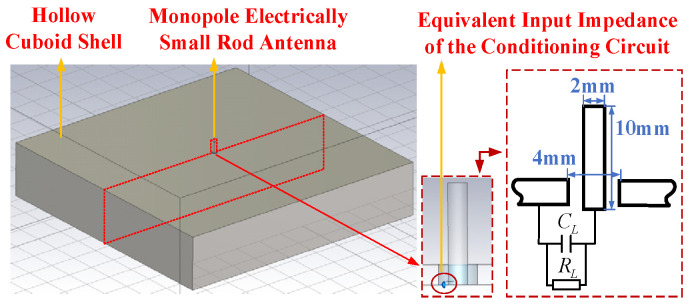
Finite element simulation model of transient E-field sensor.

To analyze the time-domain response and the amplitude–frequency characteristics in the frequency domain, a simulation model was constructed in CST Microwave Studio, as shown in Figure 2. The model features a rod antenna designed as a cylindrical structure with a height of 10 mm and a radius of 1 mm. Surrounding the rod is a hollow cuboidal shell with a thickness of 2 mm. A hole with a radius of 2 mm is created on the upper surface of the hollow shell, and the cylindrical rod is positioned inside. Both the cylindrical rod and the hollow shell are made of copper, while the remaining simulation region is set to vacuum.

Considering that the ground potential of the conditioning circuit is equal to the ground potential of the metal case in the practical use of the sensor, the designed conditioning circuit has an equivalent input impedance consisting of a 5 pF capacitor in parallel with a 1012 Ω resistor. To account for this, a lumped element is added between the pole and the metal case. The sensor features a metallic casing and an enclosed structural design to shield against external electromagnetic interference, which adopts a compact structural design to avoid interfering with the test results. The sensor’s size is 4 cm × 4 cm × 3 cm (length × width × height).

**Figure 3:**
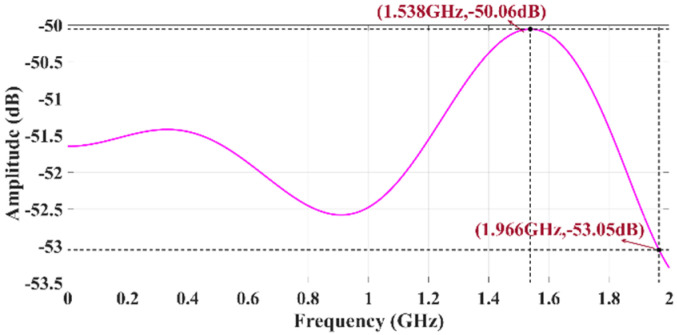
Transient E-field sensor amplitude–frequency characteristic curve.

If a Gaussian pulse waveform is used as the excitation, the amplitude–frequency response curve of the rod-shaped monopole antenna is shown in Figure 3, with a 3 dB bandwidth ranging from DC to 1.966 GHz.

### 2.3. Design of Conditioning Circuit

To maximize the electric field energy transferring to the monopole antenna, the impedance of antenna was matched to the impedance of the measurement system. The BUF802 produced by Texas Instruments was utilized to convert the high impedance to 50 ohm. The BUF802 has two operating modes, Buffer (BF) Mode and Composite Loop (CL) Mode, each suitable for different situations.

**Figure 4:**
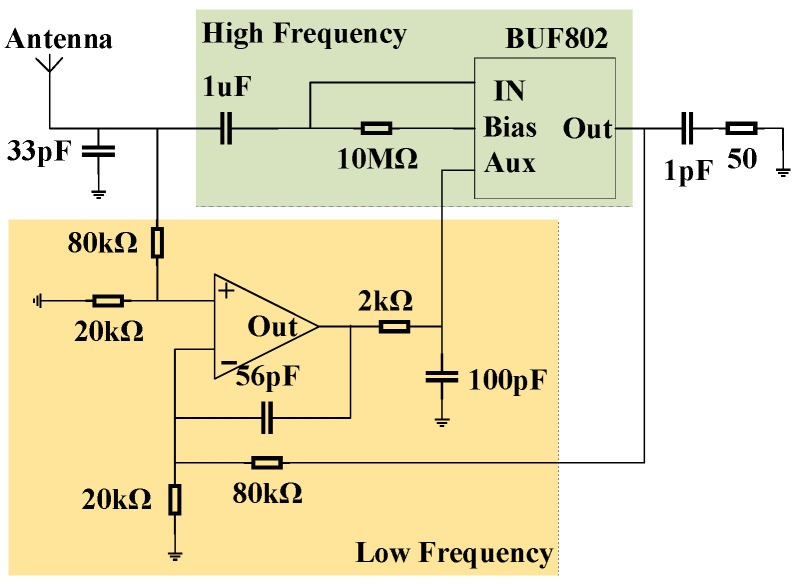
Schematic diagram of the conditioning circuit.

**Figure 5:**
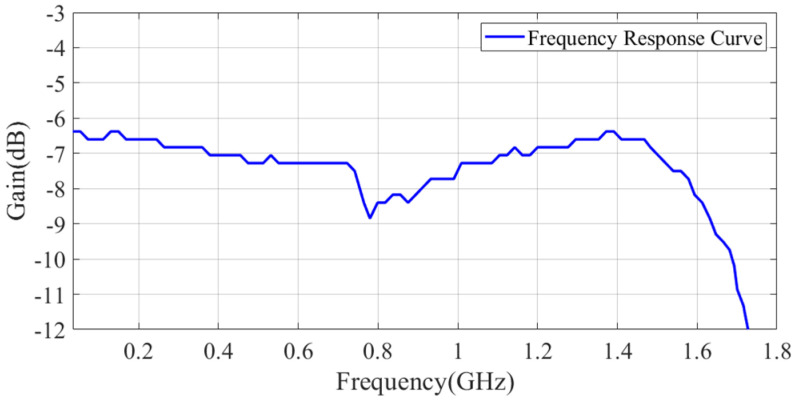
Frequency response test of the conditioning circuit by an R&S ZVA24 vector network analyzer.

In CL mode, the BUF802 features a main path and an auxiliary path. The input signal is split by a capacitor divider into high-frequency and low-frequency components. The high-frequency signal passes through the main path, while the low-frequency signal passes through the auxiliary path. These two components are then recombined at the output to form the final output signal. There are transition band segments at both low- and high-frequency ranges, where the main path and auxiliary path work together to produce the output signal. Wideband measurement requires that the amplitude–frequency response of the sensor be consistent in the low- and high-frequency ranges. If there is a difference in gain, the measurement results will show amplitude distortion. The schematic diagram of the conditioning circuit design is shown in Figure 4. A frequency sweep test was conducted on the conditioning circuit using a network analyzer, measured by the S21 parameter as shown in Figure 5. According to the frequency response curve, the measurement bandwidth of the conditioning circuit is DC to 1.65 GHz.

## 3. High-Speed Digital Acquisition System Based on FPGA

In this paper, we propose digitizing the analog signals output by the sensors at the measurement front end and then transmitting the digital signals via optical fibers. Unlike traditional oscilloscopes, the high-speed analog-to-digital conversion module in this system must also handle tasks such as analog-to-digital conversion, optical fiber communication, large data storage, and remote parameter adjustment.

**Figure 6:**
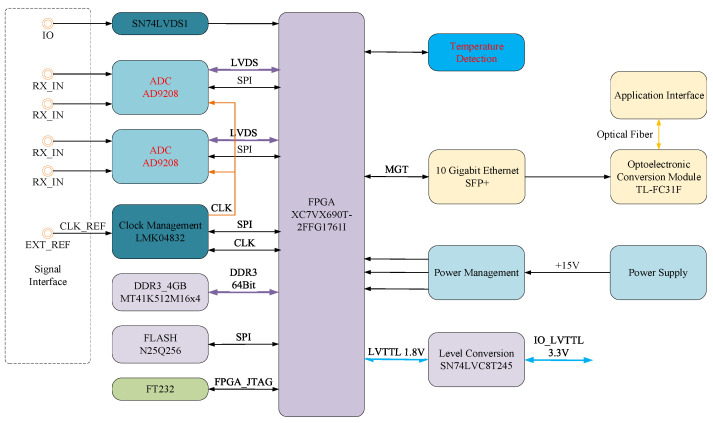
Functional block diagram of a high-speed digital acquisition system.

The system components of the high-speed digital acquisition system are shown in Figure 6. The main components include the FPGA control unit, the ADC high-speed digital sampling unit, the data buffering unit, timing generation and control, and the data communication interface.

### 3.1. Logic Control of FPGA

The FPGA chip is primarily responsible for configuring and controlling the clock chip and ADC to ensure their proper operation. The main control chip used in the broadband transient electromagnetic pulse signal acquisition system is the Xilinx Virtex-7 series XC7VX690T-2FFG1761I. This chip features 600,000 logic cells, 36 GTH pairs, and supports x8 Gen 2 PCIe interfaces. It also has 850 user I/O interfaces, making it adaptable to various application interfaces. Additionally, the FPGA includes 3100 DSP slices and supports the MIG core, enabling access to DDR3 memory, providing high data processing and buffering performance. Its main functions include the following:

1. The FPGA acquires high-speed sampled digital signals from the ADC (analog-to-digital converter) and processes these data in real-time, including operations such as data buffering, filtering, and windowing.
2. It implements the control and management of complex data streams, separating and buffering data for subsequent storage or transmission.
3. The FPGA stores the data received from the ADC into high-speed memory (such as DDR3) and transfers the data to other modules or external devices as needed—for example, transmitting to a host computer via optical fibers.
4. The FPGA is responsible for transmitting the acquired digital signals to external systems through the Quad Small Form-factor Pluggable (QSFP) interface, achieving interface control and communication protocols. The external I/O level is 3.3 V, while the FPGA’s I/O level is 1.8 V. The SN74LVDSI chip is used to achieve compatible level conversion between them.
5. Precise timing control is crucial in high-speed data acquisition systems. The FPGA generates and manages the clock signals for the entire analog-to-digital conversion circuit, ensuring synchronization between the ADC and other components.

### 3.2. High-Speed Analog-to-Digital Conversion

The analog-to-digital conversion function is primarily accomplished using high-speed ADC chips, taking into account factors such as bandwidth, sampling frequency, timing requirements, power consumption, and communication protocols. Two high-speed ADC chips, AD9208 from Analog Devices (ADI), were selected for this purpose. The AD9208 is a dual-channel, 14-bit ADC with a maximum sampling rate of 3000 MSPS. This device is optimized for a wide input bandwidth, high sampling rates, good linearity, and low power consumption in a small package. It includes on-chip buffers and a sample-and-hold circuit, designed specifically for low power, small size, and ease of use. The product is used for sampling wideband analog signals. This dual-channel ADC core employs a multi-stage, differential pipeline architecture with integrated output error correction logic. Each ADC has a wideband buffered input, supporting various selectable input ranges. The integrated reference voltage source simplifies design. The data outputs of each ADC are internally connected to an optional 1/2 decimation module.

Both the analog input and clock signals are differential input signals. The data outputs of each ADC are internally connected to two digital downconverters (DDCs). Each DDC consists of four cascaded signal processing stages: a numerically controlled oscillator (NCO) and three half-band decimation filters that support 2×, 4×, and 8× decimation.

### 3.3. Data Cache

Since the application involves capturing nanosecond pulse train signals at a high sampling rate while providing millisecond-level storage duration, the system requires a large storage capacity. To address concerns about potential memory bandwidth limitations with two 12-bit ADCs operating at 3 GS/s each, our system employs four Micron MT41K256M16 DDR3 memory chips, each with a 16-bit data width and 4 Gb capacity. Configured in parallel, they form a 64-bit wide DDR3 memory interface totaling 2 gigabytes (GB), directly connected to the FPGA for high-speed data acquisition and storage. This design ensures that our system can store and process data at the full rate generated by the ADCs, effectively mitigating concerns related to memory bandwidth limitations. FLASH memory is used to store the nonvolatile FGPA’s configuration.

### 3.4. Timing Clock Generator

The sampling clock signal frequency of our designed digital acquisition system is fixed at 3 GSps. We selected clock chips LMK04832 from Texas Instruments (TI) to provide the reference clock for the entire sampling system. The LMK04832 can provide 14 output clock signals, with a frequency range up to 3255 MHz, which provides two 3 GHz clock signals to the two AD9208 chips. Additionally, by using frequency division and working in conjunction with the FPGA, the clock signals are distributed to the data storage, communication interface, and other modules.

### 3.5. Data Communication

The FPGA transmits the acquired digital data at high speed through QSFP, with the digital signals transmitted via optical fibers. Each channel typically achieves a transmission rate of 10 Gbps or even higher. The optical signals reach the remote host computer and are converted back into electrical signals, which are then transmitted through the network interface to the host computer for display and control. Additionally, the host computer can send control commands via optical fibers to configure the parameters and trigger, reset, and control the acquisition system.

## 4. Calibration for the Electric Field Sensor

### 4.1. Calibration for Conversion Factor Curve

Typically, the electric field *E* in space is measured using an electric field sensor, which outputs a voltage signal *V*. It is necessary to determine the proportionality factor *K* = *V*/*E* and evaluate the linearity of *K* under different electric field intensities. This paper conducts sensor calibration based on a TEM cell which is also known as a transverse electromagnetic wave chamber [27,28]. It is constructed with the outer conductor’s middle section, formed by joining the top and bottom plates, while the inner conductor’s middle section is a strip-shaped partition. The ends of the partition gradually taper and connect to the inner conductor of a coaxial cable with a characteristic impedance of 50 Ω. This configuration forms a main transmission section in the middle and horn-shaped transition sections at both ends. A square voltage signal is injected into one end, whose rising time is about 800 ps, corresponding to an upper frequency limit of $f_{H}=\frac{0.35}{t_{r}}=437\;MHz$. Then, a uniformly propagating transverse electromagnetic wave is generated within the TEM cell.

**Figure 7:**
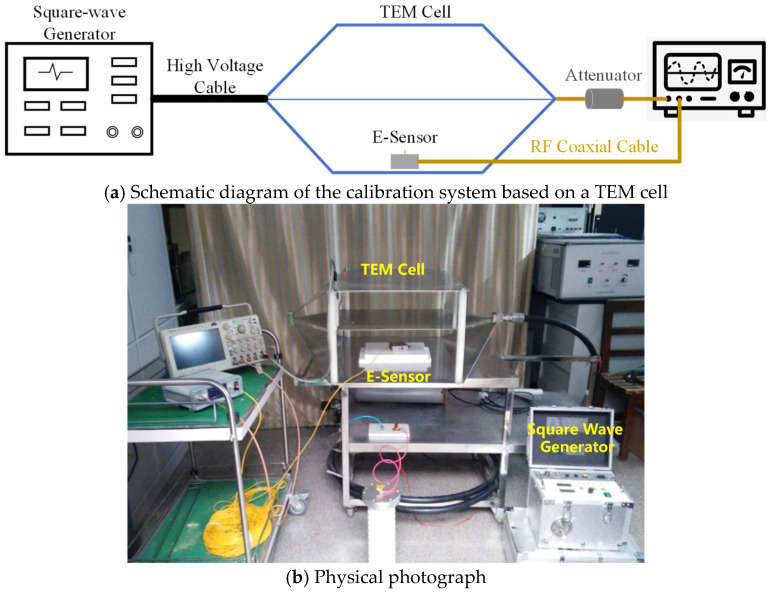
Principle of transient electric field sensor calibration based on a TEM cell.

**Figure 8:**
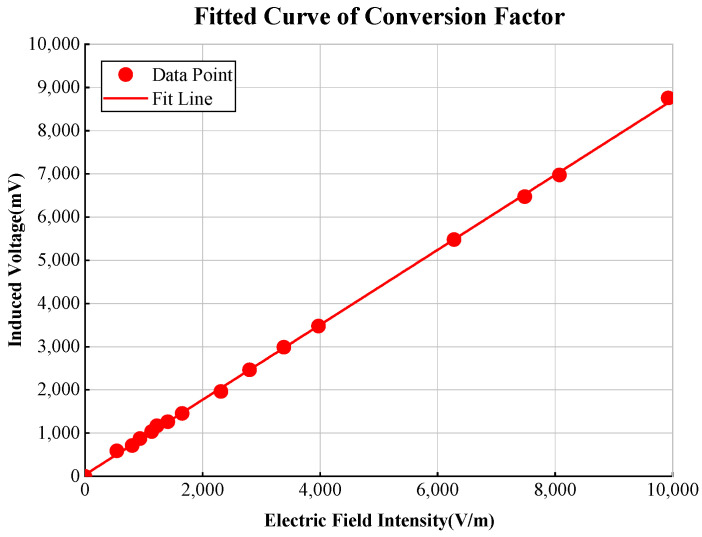
Fitted curve of the conversion factor.

Using the TEM cell structure in Figure 7 as an example, the input port is connected to the pulse source via a coaxial cable, and the output port is connected to an oscilloscope. Once the conversion factor K is determined, the numerical coefficient between the output voltage and the input electric field can be achieved. The fitted curve of conversion factor is shown in Figure 8, which shows good linearity. The slope of the fitted line represents the conversion factor K, which is approximately 0.87 mV/(V/m).

**Figure 9:**
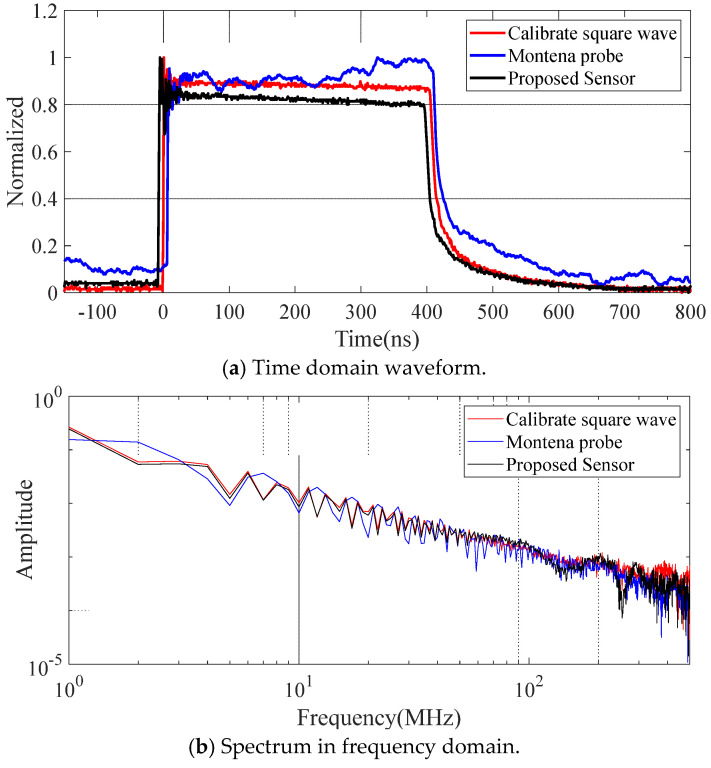
Measured waveform of the proposed sensor and D-dot antenna in TEM Cell.

A D-dot antenna (SGE3-5G from Montena Inc.) and the proposed monopole antenna are selected for calibration in the TEM cell, as shown in Figure 9. In the time-domain waveform, the D-dot antenna exhibits local oscillations in the high-level flat region of the square wave, indicating poor low-frequency response. This observation is further confirmed in the frequency-domain analysis, where the spectrum of D-dot shows obvious differences compared with the reference square wave. In comparison, the proposed sensor demonstrates good consistency with the calibrated square wave in both the time-domain waveform and the frequency spectrum, highlighting its superior performance.

### 4.2. Calibration for High-Frequency Response

**Figure 10:**
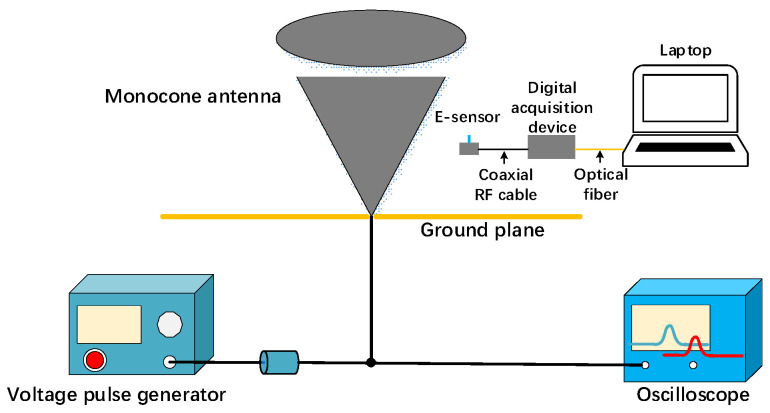
Experimental configuration of time domain calibration based on single cone antenna.

**Figure 11:**
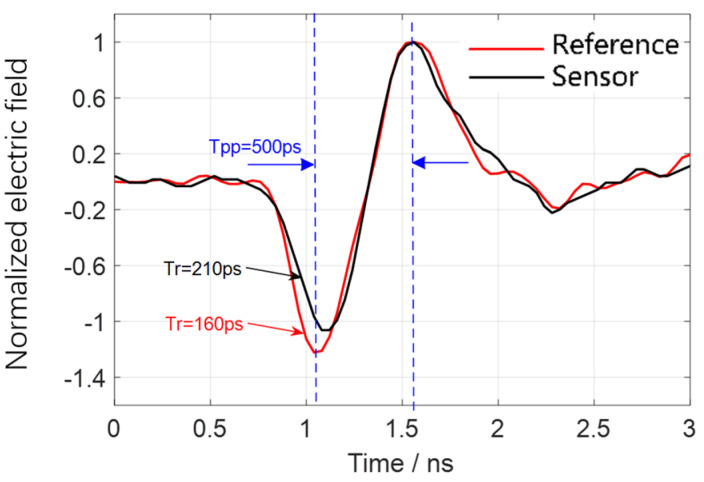
Calibration measured waveform in time domain.

However, the calibration by the TEM cell is insufficient to evaluate the system’s upper frequency limit of 1.6 GHz. Therefore, a monocone antenna is employed to test the system’s time-domain response capabilities at its upper frequency limit. The time-domain calibration of the high-frequency bandwidth response characteristics of the sensor based on the monocone antenna was implemented as described in detail in [29,30]. The configuration of the calibration experiment is shown in Figure 10. The reference waveform used in the experiment is a bipolar pulse with a rise time of *Tr* = 160 ps and a positive-negative pulse delay of 500 ps, which is shown in Figure 11. In comparison, the rise time of the electric field signal measured by the sensor is 210 ps, which is slightly longer than the rise time of the reference pulse. The corresponding upper cutoff frequency is approximately 1.66 GHz, consistent with the frequency sweep curve of the sensor shown in Figure 5.

## 5. Site Testing in HVDC Station

**Figure 12:**
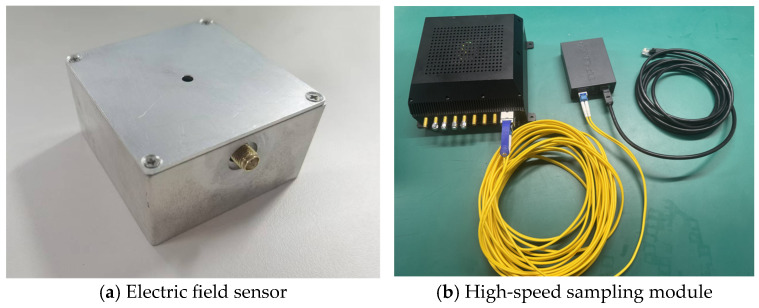
Calibration measured waveform in time domain.

Based on the principles described above, the electric field sensor is shown in Figure 12a, the outline size of the high-speed sampling module is 156 mm × 166 mm × 72 mm, and the front-end sensor can be flexibly replaced. The final physical picture is shown in Figure 12b.

To verify the performance of the electric field measurement system, on-site tests were conducted at the ±400 kV Qaidam Converter Station in Qinghai Province, China. The radiated electric field was measured under two conditions: during the operation of the GIS disconnect switch and during stable operation near the AC busbar.

### 5.1. Disconnect Switch Operation Test

**Figure 13:**
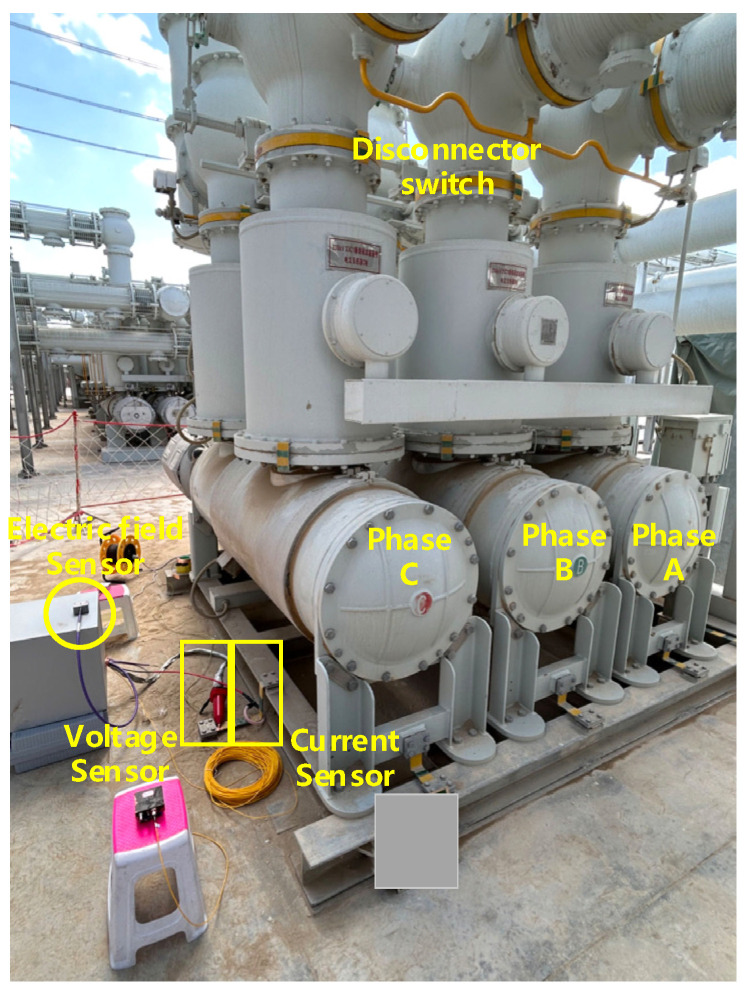
On-site equipment configuration for the disconnect switch opening test.

During the power outage maintenance process at the converter station, it is necessary to segment and disconnect two 350 kV busbars, involving the operation of multiple GIS circuit breakers and disconnect switches. Research has shown that the GIS circuit breaker contains an SF6 arc extinguishing chamber, which has a certain ability to suppress over-voltages caused by breaking unloaded lines. Therefore, the probability of overvoltage events caused by arc reignition during circuit breaker operation is considered lower than that during disconnect switch operation. When the disconnect switch is opened, due to its poor arc extinguishing performance, the opening operation can cause multiple arc reignitions in the contact gap, forming very fast transient overvoltages (VFTOs) that are coupled to the GIS enclosure and grounded, generating a transient ground potential rise. Simultaneously, transient grounding currents arise, and transient electromagnetic fields are coupled into the surrounding space, as detected by our sensor. The transient electric field pulse signals generated by GIS switch operations can have extremely fast rise times, reaching the nanosecond or picosecond level, covering a wide frequency spectrum from a few kilohertz (kHz) to several gigahertz (GHz). This test scenario is one of the typical applicational scenarios in electrical equipment fault diagnosis, and it can be used to evaluate the performance of the proposed sensor as well. The electric field sensor is placed about 1 m from the disconnect switch, at a height of approximately 0.5 m above the ground. The voltage and current sensor are connected to the digital acquisition system at the same time. All sensors are connected to a high-speed digital acquisition system inside a shielded box via a high-frequency shielded coaxial cable. So, it is possible to measure the electric field, voltage, and current simultaneously. The experimental setup is shown in Figure 13, whose parameters are shown in Table 1.

**Figure 14:**
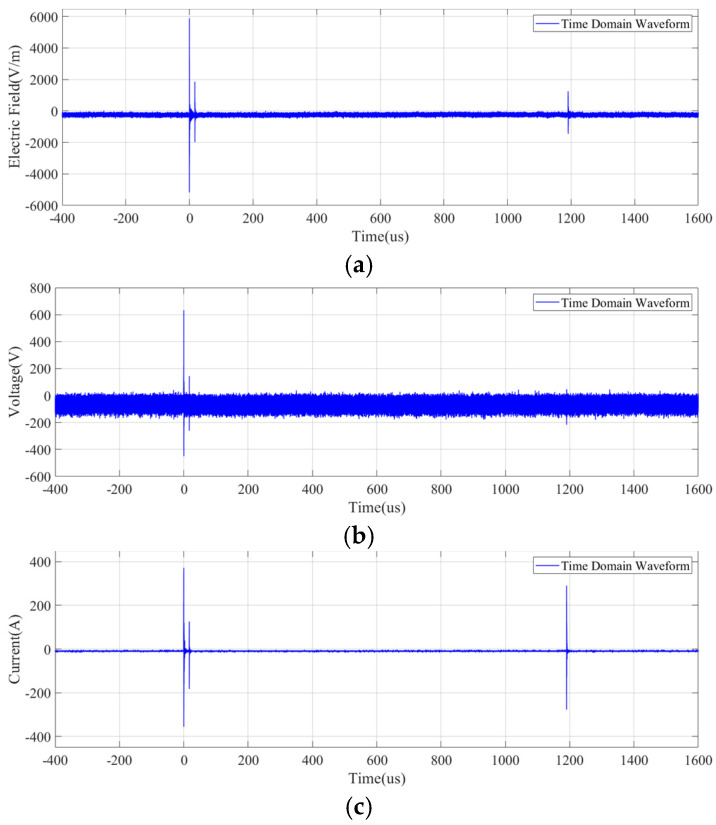
Measured switch field pulse train signal in time domain: (**a**) electric field; (**b**) voltage; (**c**) current.

**Figure 15:**
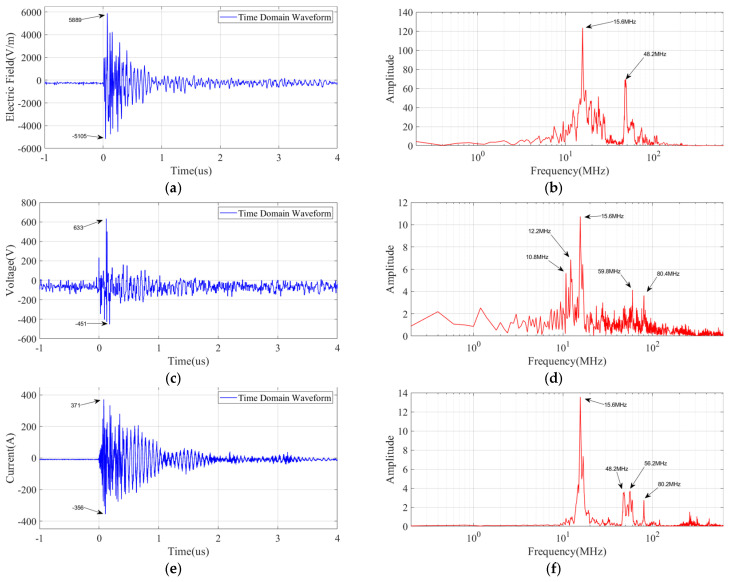
Detail of the pulse signal generated by switch operation: (**a**) electric field in the time domain; (**b**) electric field in the frequency domain; (**c**) voltage in the time domain; (**d**) voltage in the frequency domain; (**e**) current in the time domain; (**f**) current in the frequency domain.

From Figure 14, it can be observed that the time-domain waveforms of the electric field, voltage, and current all exhibit similar pulses with identical occurrence times. The three pulse times are 0 μs, 17 μs, and 1190 μs, which reflect the reignition moments of the arc. Figure 15 shows the local detailed time-domain waveform and corresponding spectrum of the pulse at the moment of the opening operation. The electric field appears as a pulse train with a damped oscillatory wave shape, reaching a maximum amplitude of 5.65 kV, with a pulse rise time of 12 ns and a decay time of approximately 1 μs. The dominant spectral components of the electric field signal are 15.6 MHz, being the same with the voltage and current, whose upper frequency components are over 80 MHz.

This test confirms that the sensor possesses sufficient response speed and bandwidth to effectively measure the nanosecond electromagnetic transient such as electric field, voltage and current signals simultaneously.

### 5.2. Power Frequency Steady-State Electric Field Test

**Figure 16:**
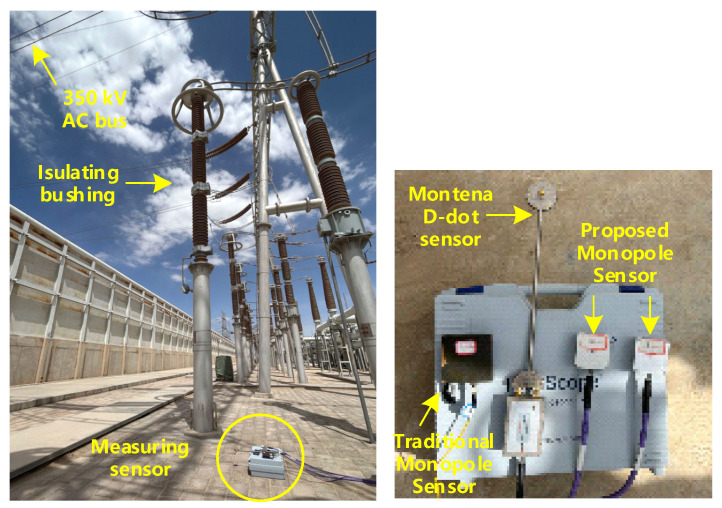
On-site equipment configuration for the steady-state test.

To test the low-frequency performance of our designed monopole antenna, we measured the steady-state electric field beneath an AC overhead busbar. To compare the response of different antennas at the 50 Hz power frequency, the measurement included a D-dot antenna from Montena Inc., a traditional monopole sensor, and our designed ultra-wideband monopole sensor. The layout of the experiment is shown in Figure 16.

**Figure 17:**
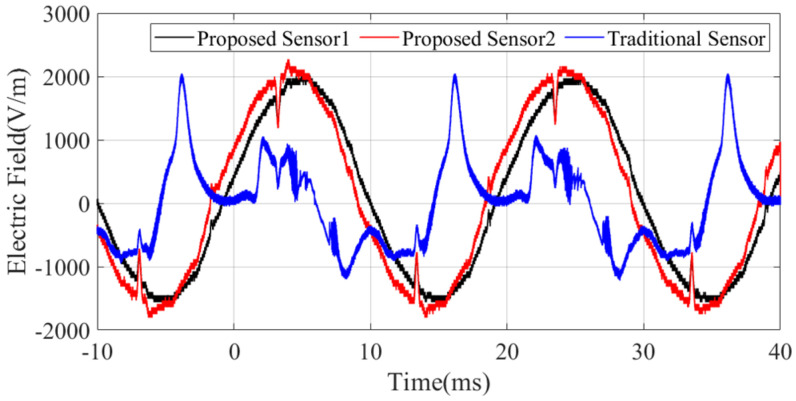
The waveform of the power frequency induced electric field signal.

**Figure 18:**
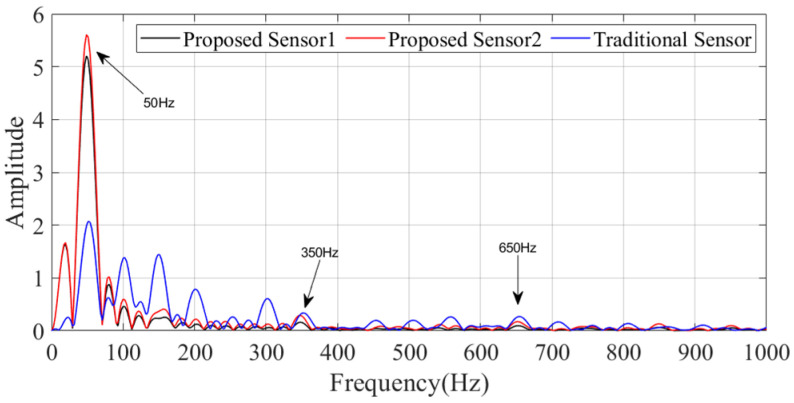
The spectrum of the power-frequency-induced electric field signal.

The measured electric field waveform is shown in Figure 17, and the spectrum is shown in Figure 18. The blue curve represents the measurements performed by the traditional monopole sensor, which exhibits significant waveform distortion and only reflects a repeating period of approximately 20 ms. The red and black curves represent the waveforms measured by our designed ultra-wideband monopole sensor, which more accurately reflect the waveform of the power frequency voltage with a repeating frequency of 50 Hz. This is because the conditioning circuit of the electric field sensor adopts a split-band matching design for high and low frequencies, resulting in a flatter frequency response curve in the low-frequency region.

Notably, there are spikes of opposite polarity superimposed at specific phases of the power frequency waveform, which may be caused by high-frequency pulses from corona discharges on the busbar. From the spectrum, apart from the fundamental frequency of 50 Hz, they all contain some high-frequency components, such as 350 Hz and 650 Hz.

In comparison, the D-dot antenna from Montena Inc does not measure an effective electric field waveform, because the coupled electric field from the power frequency voltage exceeds the lower frequency limit of the sensor.

## 6. Conclusions

To address the current shortcomings of transient electric field sensors, such as poor low-frequency performance, narrow bandwidth, insufficient accuracy, large size, unstable amplitude coefficients, and low system integration, we propose a new technical approach based on FPGA high-speed digital sampling and optical fiber communication. To design the sensor with electromagnetic interference resistance, we employed front-end high-speed analog-to-digital technology to sample the analog signal and used optical fibers to transmit the digital signal. This approach maximizes the integrity of signal while achieving electromagnetic interference resistance, and it effectively improves the sensitivity and coefficient stability of the sensor.

Based on the working principle of the rod-shaped electrically small antenna, we studied and optimized the design of the conditioning circuit for the induced voltage of the electric dipole antenna, extending the sensor’s analog bandwidth from the original 1 kHz to 460 MHz to an ultra-wide bandwidth from DC to over 1.6 GHz.

On-site validation tests were conducted at the ±400 kV Qaidam Converter Station in Qinghai Province, where we measured the transient electric fields generated by disconnect switch operations as well as the steady-state power frequency electric fields under normal operating conditions. A comparative analysis of the performance of different antenna sensors showed that our designed sensor outperforms the D-dot and traditional monopole antenna sensors in low-frequency scenarios. The proposed sensor successfully captured the coupled electric field waveform at the power frequency (50 Hz), with its waveform matching the period of the power frequency voltage, in accordance with the quasi-electrostatic field theory. In comparison, the D-dot antenna failed to detect this low-frequency signal due to noise interference, while the waveform of the traditional sensor exhibited significant distortion.

This study demonstrates that the electric field measurement capabilities of the sensor cover a range from power frequency steady-state signals to VFTO and high-frequency transient signals such as partial discharges. This measurement system shows great potential in the fields of condition monitoring, fault detection, and diagnostics, as well as in partial discharge fault localization in power equipment.

## Figures and Tables

**Table 1:** The indicators of measuring equipment.

Sensor	Bandwidth	Measurement Range
Voltage Sensor	220 MHz	27 kV
Current Sensor	16 MHz	30 kA

## Data Availability

Data are unavailable due to privacy restrictions.

## Abbreviations

The following abbreviations are used in this manuscript:

FPGA	Field-programmable gate array
EMP	Electromagnetic pulse
HEMP	High-altitude electromagnetic pulse
IGBT	Insulated gate bipolar transistor
ADC	Analog-to-digital converter
TEM	Transverse electromagnetic
GIS	Gas insulated switchgear
VFTO	Very fast transient overvoltage
